# Systematic Performance Evaluation of a Novel Optimized Differential Localization Method for Capsule Endoscopes †

**DOI:** 10.3390/s21093180

**Published:** 2021-05-03

**Authors:** Samuel Zeising, Kivanc Ararat, Angelika Thalmayer, Daisuke Anzai, Georg Fischer, Jens Kirchner

**Affiliations:** 1 Institute for Electronics Engineering, Friedrich-Alexander-Universität Erlangen-Nürnberg (FAU), 91058 Erlangen, Germany; kivanc.ararat@fau.de (K.A.); angelika.thalmayer@fau.de (A.T.); georg.fischer@fau.de (G.F.); jens.kirchner@fau.de (J.K.); 2 Department of Electrical and Mechanical Engineering, Nagoya Institute of Technology, Nagoya 466-8555, Japan; anzai@nitech.ac.jp

**Keywords:** geomagnetic compensation, magnetic localization, wireless capsule endoscopy (WCE)

## Abstract

Capsule endoscopy is a well-established diagnostic tool for the gastrointestinal tract. However, the reliable tracking of capsule endoscopes needs further investigation. Recently, the static magnetic differential method for the localization of capsule endoscopes has shown promising results. This method was experimentally validated by investigating the difference in the measured values of the geomagnetic flux density of a representative sensor pair. In the measurements, it was revealed that misalignment of the sensors and ferromagnetic material near the sensor pair had the most significant impact on the differential approach. Besides, a systematical simulation-based study was conducted. Herein, the position and alignment of all sensors of the localization system were randomly varied. Furthermore, root-mean-squared noise was added to the sensor measurements, and the influence of nearby ferromagnetic material was evaluated. Subsequently, non-idealities were applied simultaneously on the proposed localization system, and the entire system was rotated. The proposed method was significantly better than state-of-the-art geomagnetic compensation methods for the localization of capsule endoscopes with mean position and orientation errors of approximately 2 mm and 1°, respectively.

## 1. Introduction

Over the past 20 years, the medical application of wireless capsule endoscopy (WCE) for the diagnosis of the gastrointestinal tract (GIT) has been rapidly developed [1,2,3]. Compared with traditional endoscopy, WCE can be applied with less traumatic procedures for the patient. The complete diagnosis with WCE takes up to 12 h [4]. Although WCE has a crucial role in obtaining useful images of the GIT, particularly from the diseased region, identifying the exact location of the detected disease is still an open research problem. Therefore, the precise localization of the capsule and detected disease during diagnosis is of particular interest among researchers in the field of WCE.

Various approaches have been utilized for the localization of capsule endoscopes. The three most promising methods are the video-, radio-frequency- (RF) and magnetic field-based methods [3,5].

The first localization method uses the video captured by the WCE to obtain information about the relative changes between consecutive video frames. Because image processing for localization can be done on an external computer, no additional hardware is needed inside the capsule. However, when the video-based localization method is used alone, the localization performance is insufficient [3,6]. Therefore, the video-based localization method is generally combined with the RF-based or magnetic-based localization method to obtain additional information, i.e., the rotation of the capsule around the longitudinal axis [7,8] or the speed of the capsule [9], to enhance the overall tracking process of the capsule.

The second method is based on conventional RF-tracking methods (e.g., received signal strength (RSS) [10] and time of arrival (TOA) [11,12]). Herein, the propagation properties of an electromagnetic wave are utilized to localize the capsule endoscope. Geng et al. (2016) [13] conducted a comprehensive study of the posterior Cramer–Rao lower bound using the RSS and TOA approaches for the localization of WCE. They determined that accuracy in the mm-range is theoretically achievable when the RF-based localization method is combined with the video-based localization method. Ito et al. [14] proposed a hybrid RF method where the average relative permittivity of a human body phantom was estimated. Subsequently, this information was used to locate the capsule with the TOA-method. In this way, accuracy in the mm-range was achieved. However, in the aforementioned studies, the human body phantom was simplified. Because the propagation parameters of the human body vary significantly for the different layers of tissues and different patients, achieving localization accuracy in the mm-range with the RF-based method in real measurements is very challenging.

The third method is the magnetic field-based localization of WCE capsules, which showed the best localization performance [3,5]. Well-established magnetic localization methods are either based on transmitting a magnetic signal using a coil [15,16,17,18,19] or a permanent magnet [4,20,21,22,23]. Permanent magnets as a magnetic field source are integrated into the capsule. The generated static magnetic field is sensed by a magnetic sensor array on the body surface of a patient. When a coil is used as a magnetic field source, a receiver coil [15] or magnetometer [18] is integrated into the capsule. When a coil is used as a magnetic field source outside the body, the number of sensors/receiving coils is restricted due to the limited space within the capsules. Since magnetic field-based localization is based on solving a non-linear equation system, the number of sensors and, therefore, equations is crucial for the localization accuracy. By integrating a permanent magnet into a capsule for WCE, promising localization results have been achieved so far. Since it is a passive magnetic field source, capsule batteries are not required for the localization process. Moreover, a larger number of sensors can be placed outside the body.

However, on the body surface, the magnetic flux density of the permanent magnet is of the same order as the geomagnetic flux density and, thus, leads to high localization errors [24]. A straightforward approach to address this issue is to calibrate the magnetic sensor array according to the geomagnetic field [20,22]. However, this procedure is only valid as long as there is no relative rotation between the sensors and the Earth. Because the entire WCE procedure takes several hours, sensor calibration is not practicable as a patient wearing the sensor system cannot be expected to keep still during the entire examination.

Shao et al. (2019) [4] proposed a novel static magnetic localization method for capsule endoscopes to prevent the interference of the geomagnetic field. A wearable localization system consisting of 16 magnetic sensors with two additional magnetic sensors for geomagnetic compensation was applied. The additional sensors were mounted on the back and chest of a patient. Because of the considerable distance from the intestine, the two additional sensors were assumed to measure only the geomagnetic flux density. Therefore, by subtracting the flux density of the two additional sensors from the measured values of the 16 sensors, the geomagnetic flux density was canceled. However, the orientation error varied significantly when the localization setup was rotated.

Dai et al. (2019) [25] applied an inertial sensor-based geomagnetic compensation approach for magnetic tracking of a capsule endoscope. The inertial sensor was used to separate the three components of the geomagnetic field from the permanent magnet field. Although this approach is useful to remove geomagnetic components from the permanent magnet field, it still uses an inertial sensor, which suffers from drift error over time. In Dai’s study, the localization system stability was tested only for a short time interval of approximately 90 s. This is particularly problematic if the relatively long duration of a diagnosis with WCE is considered.

In our previous work [26], we optimized our novel differential static magnetic localization method with geomagnetic compensation and evaluated it in numerical simulations. The proposed method was based on a localization system, consisting of three stable rings with four magnetic sensors mounted on each ring. The sensors were grouped into pairs, consisting of identically orientated sensors, and by subtracting the measured values of those sensors, the geomagnetic flux density was cancelled out. The main goal of our proposed method was to enable a patient to leave the hospital during the relatively long diagnosis. Therefore, the intended use-cases of the proposed differential localization method for capsule endoscopes are daily life situations of a patient. Therefore, in this study, we investigated the feasibility of the proposed method under more realistic scenarios, which represent daily life activities. The non-idealities of the localization system, such as misalignment and displacement, as well as the root-mean-squared (RMS)-noise of the sensors were considered. Moreover, the influence of the ferromagnetic material on localization performance was investigated. Besides, the entire system was rotated while the resulting localization accuracy was determined.

## 2. Fundamentals of Static Magnetic Localization

### 2.1. Magnetic Dipole Model

**Figure 1:**
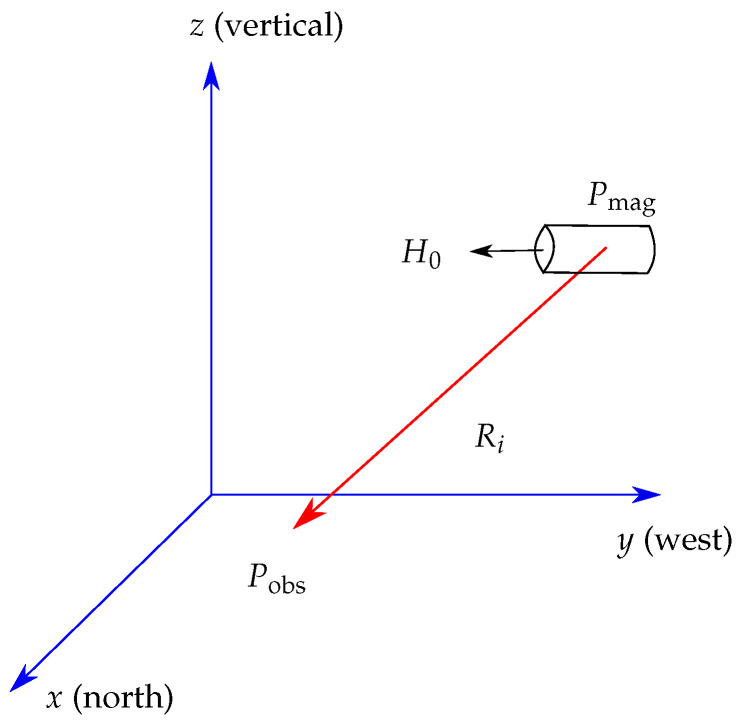
Localization scenario of a permanent magnet with its reference coordinate system.

A cylindrical permanent magnet (Figure 1) at position $P_{\mathrm{mag}}$, which has a length *l*, radius *k*, and magnetization $M_{0}$ in amperes per meter, generates a magnetic flux density $B_{\mathrm{mag}}$ in teslas. For this scenario and in the case that the Euclidean distance ${∥R∥}_{2}$ between $P_{\mathrm{mag}}$ and an observer point $P_{\mathrm{obs}}$ is much larger than the geometry of the magnet, $B_{\mathrm{mag}}(P_{\mathrm{obs}})$ can be analytically expressed by the magnetic dipole model [27]:

(1) $$B_{\mathrm{mag}}\left(P_{\mathrm{obs}}\right)=\frac{\mu_{0}\mu_{r}M_{0}l\pi k^{2}}{4\pi }\left(\frac{3\langle H_{0},R\rangle R}{{∥R∥}_{2}^{5}}-\frac{H_{0}}{{∥R∥}_{2}^{3}}\right).$$

The orientation vector of the permanent magnet is expressed by $H_{0}$. The magnetic permeability in a vacuum is $\mu_{0}$ = 4 π × $10^{-7}$ V s $A^{-1}$ $m^{-1}$, and the relative permeability $\mu_{r}$ of human tissue is ≈1 [28].

### 2.2. Soft Magnetic Distortion

**Figure 2:**
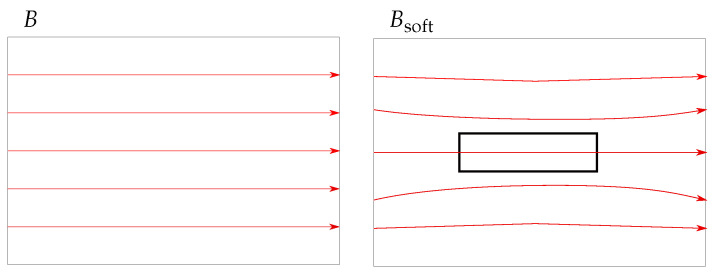
Comparison of the magnetic flux density *B* for undistorted (**left**) and soft-iron distorted (**right**). A ferromagnetic material (black rectangle) is placed within *B*.

Soft magnetic distortion influences existing magnetic fields (e.g., the geomagnetic field or the magnetic field generated by a magnet) and is caused by ferromagnetic objects. In the proximity of the ferromagnetic material, the geomagnetic field can no longer be assumed homogeneous (Figure 2). Magnetic sensors that measure the geomagnetic field must be calibrated concerning soft magnetic distortions. However, the calibration is only valid for a static scenario (no relative movement between the ferromagnetic material and a sensor).

## 3. Methods

### 3.1. Localization Setup

**Figure 3:**
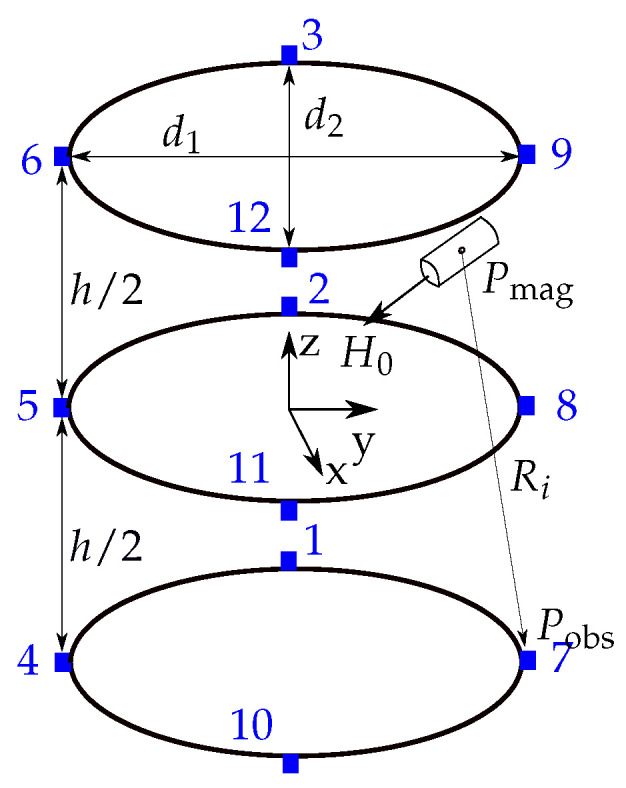
Localization scenario of a permanent magnet with the proposed localization system and the reference coordinate system.

To estimate the position and orientation of a magnet, the localization setup proposed in [24] was considered. Three identical, stable, and elliptical rings with a large diameter of $d_{1}=40$ cm and a small diameter of $d_{2}=33$ cm were established with four magnetic sensors mounted on each ring, leading to 12 sensors in total (Figure 3). The distance h/2 between one ring and another ring was 10 cm. Hence, the sensor setup is suitable for a wearable localization setup for the abdomen of a subject. The reference coordinate system for the localization of the magnet has its origin in the center of the middle sensor ring. The diameter, as well as the length of the neodymium magnet were both set to 10 mm, and its axial magnetization was assumed to be 1150 kA/m.

The three components of the magnetic flux density ${\left({\hat{B}}_{x_{i}},{\hat{B}}_{y_{i}},{\hat{B}}_{z_{i}}\right)}^{⊺}$ were measured by using the *i*th sensor. In the following, $\hat{B}=B_{\mathrm{mag}}+B_{\mathrm{geo}}$ and *B* indicate the measured and analytical magnetic flux densities, respectively. The geomagnetic flux density $B_{\mathrm{geo}}$ with the *x*- (north), *y*- (west), and *z*- (vertical) components for Erlangen, Germany, were assumed to be approximately ${\left(20.2,-1.2,-44.5\right)}^{⊺}$ µT [29] for this study.

### 3.2. Simulation Setup

**Figure 4:**
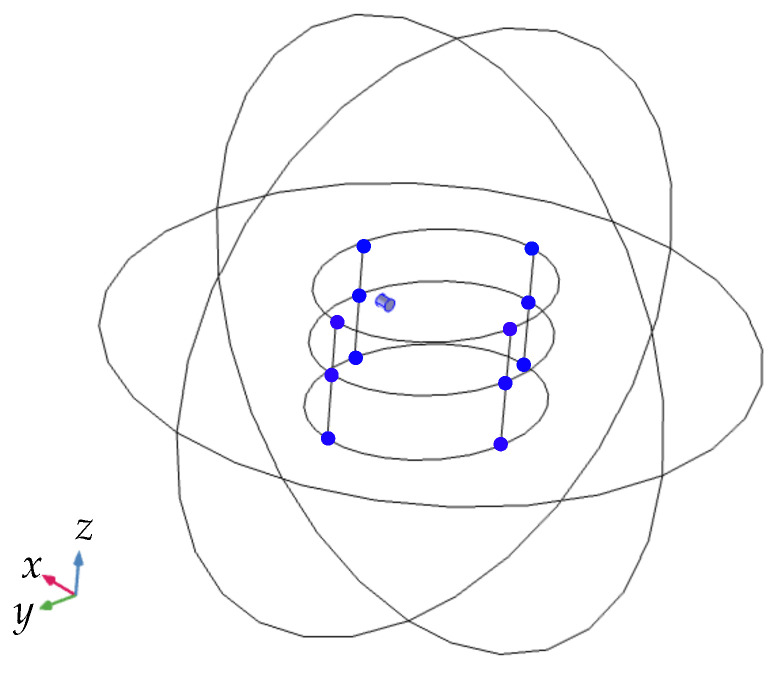
Proposed simulation setup in COMSOL Multiphysics^®^ 5.4. The 12 sensors and the magnet are highlighted in blue. Moreover, the spherical computational domain is shown.

The differential static magnetic localization setup was simulated in COMSOL Multiphysics^®^ 5.4. The simulation model of the localization setup described in Section 3.1 is depicted in Figure 4.

The computational domain was assumed to be a sphere with a radius of 800 mm, filled with air. This assumption is true since human tissue is non-magnetic. The radius of the sphere was determined by convergence tests concerning the localization error [26]. As a boundary condition of the computational domain, magnetic insulation (B·n=0) was applied. The permanent magnet was homogeneously axially magnetized with a magnetization of 1150 kA/m. The homogeneous geomagnetic flux density according to Section 3.1 was applied. At the 12 sensor positions, the three components of the magnetic flux density were evaluated and exported to MATLAB 2019a, where the localization algorithm was applied to the data.

#### Absolute Magnetic Localization Method

The absolute static magnetic localization method for WCE is well-established. Herein, the three respective components of the measured $\hat{B_{i}}$ were directly subtracted from those of the analytical $B_{i}$, which were derived according to Equation (1), leading to three non-linear equations per sensor. The position and orientation of a capsule can be described with 6 parameters. Thus, for the proposed localization system, a 36×6 non-linear equation system was derived, solved for the position and orientation of the magnet by minimizing the corresponding error function $\epsilon_{\mathrm{abs}}$:

(2) $$\epsilon =\sum_{i=1}^{12}{∥B_{i}-\hat{B_{i}}∥}_{2}$$

by using the Levenberg–Marquardt (LM) algorithm [30,31]. The corresponding starting vector *x* of the iterative algorithm was set to zero. Since the geomagnetic flux density is of the same order as the measured flux density generated by the integrated magnet, the localization performance by applying the absolute method is significantly affected [32].

### 3.3. Differential Localization Method

**Figure 5:**
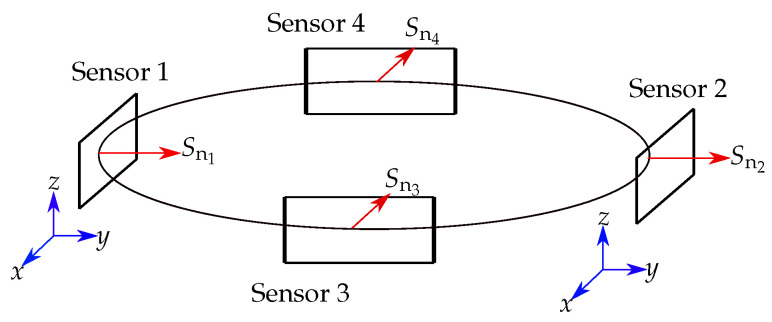
A representative sensor ring with 4 mounted sensors. The normal vector $S_{n}$ for each sensor is depicted. The coordinate systems of sensors corresponding to a pair (Sensor 1 and Sensor 2) are shown in blue.

To prevent the interference of the geomagnetic field, we proposed a differential localization method [32]. Thus, the 12 sensors of our localization setup proposed in [24] were divided into 6 sensor pairs, consisting of two identically oriented sensors (for example, Sensors 1, 2 and 3, 4 in Figure 5). The two measured values $\hat{B_{i}}$ of these pairs were subtracted from each other, leading to $\Delta \hat{B_{i}}$. For the differential method, it is crucial that the coordinate systems of the individual sensors have the same orientation as the reference coordinate system. Consequently, the normal vectors $S_{n_{i}}$ of two sensors of a pair must have the same direction and must not be rotated concerning $S_{n_{i}}$.

To estimate the position and orientation of the magnet, for each sensor pair, the derived $\Delta \hat{B_{i}}$ were subtracted from those of the analytical $\Delta B_{i}$, leading to three non-linear equations per sensor pair. Thereby, a 18×6 non-linear equation system was derived, which was solved by minimizing the error function $\epsilon_{\mathrm{diff}}$:

(3) $$\epsilon =\sum_{i=1}^{12}{∥\Delta B_{i}-\Delta \hat{B_{i}}∥}_{2}$$

The minimization of $\epsilon_{\mathrm{diff}}$ was conducted in the same way as for the absolute method. Under the assumption that a pair of sensors is perfectly aligned and calibrated as previously mentioned, the corresponding components of $B_{\mathrm{geo}}$ were identical and canceled out by applying the differential method. The localization of a capsule requires the estimation of six unknowns; since the non-linear equation system is over-determined, the differential method is valid for the proposed localization system.

### 3.4. Experimental Validation of the Differential Method

**Figure 6:**
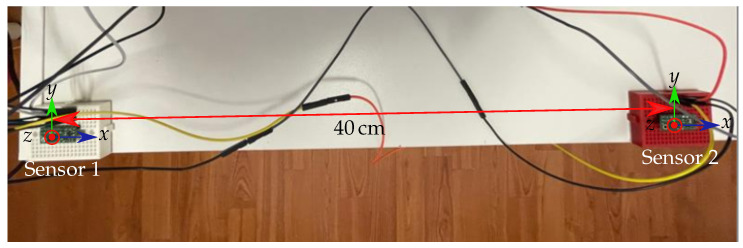
Measurement setup of a representative sensor pair. Sensors 1 and 2 are approximately equally orientated.

The feasibility of the proposed differential geomagnetic compensation method was experimentally validated. Therefore, two LSM303D magnetic sensors were connected to an Arduino UNO and simultaneously calibrated concerning hard and soft distortion by applying the ellipsoid fitting algorithm in MATLAB, proposed in [33] on the measured values. The full-scale range of the sensors was set to ±200 µT, resulting in a resolution of 8 nT/LSB. The sampling frequency was set to 25 Hz. The orientation of the two sensors was adjusted such that their coordinate systems were approximately equally orientated (Figure 6). The distance between the two sensors was approximately 40 cm and, therefore, represented a sensor pair of the proposed localization setup.

The mean values over ten samples were derived for the three measured components of the static geomagnetic flux density for both sensors. Subsequently, the values were compared to evaluate whether the geomagnetic flux density can be approximated as homogeneous for the proposed localization setup. To validate this, at first, a reference measurement was conducted, which means sensors were aligned, and no displacement was conducted. Moreover, no ferromagnetic object was in the proximity of the setup. Next, Sensor 2 was displaced at 5 mm in the *x*-direction and compared with the reference measurement of Sensor 1. Furthermore, Sensor 2 was rotated around the *z*-axis by approximately 5° to study the effect of the misalignment of a sensor pair. Finally, the setup with the two sensors was placed next to a ferromagnetic heating element with a size of 55 cm × 80 cm × 15 cm (height × length × depth) in such a way that Sensor 2 was 50 cm away from it, and the distance between the two sensors remained unchanged. The length of the heating element was approximately aligned with the *y*-axis of the sensors.

### 3.5. Position and Orientation Errors

To evaluate the performance of the localization, the position $P_{\mathrm{err}}$ and orientation $O_{\mathrm{err}}$ errors were derived from the final solution vector *x* as:

(4) $$\begin{array}{cc} P_{\mathrm{err}} & ={∥P_{\mathrm{mag}}-{\hat{P}}_{\mathrm{mag}}∥}_{2} \end{array}$$

(5) $$\begin{array}{cc} O_{\mathrm{err}} & =\arccos\left(\frac{\langle H_{0},{\hat{H}}_{0}\rangle }{{∥H_{0}∥}_{2}\cdot {∥{\hat{H}}_{0}∥}_{2}}\right), \end{array}$$

where $P_{\mathrm{err}}$ is the distance from the true position $P_{\mathrm{mag}}$ of the permanent magnet to the estimated ${\hat{P}}_{\mathrm{mag}}$. Furthermore, the angle between the real orientation vector of the magnet $H_{0}$ and the estimated one ${\hat{H}}_{0}$ is expressed by $O_{\mathrm{err}}$.

### 3.6. Differential Localization Method for Ideal Conditions

The performance of the absolute and differential methods under ideal conditions and interference of the geomagnetic field was compared in detail in [32]. Ideal conditions mean that sensor positions are exactly known, sensors are perfectly aligned and calibrated, and no ferromagnetic material is in the proximity of the system. This study [32] revealed that the differential method is significantly better than the absolute method in the case of a rotating localization system. The mean position and orientation errors were reduced by three orders in magnitude to approximately 0.05 mm and 0.05°, respectively. Moreover, the impact of the different orientations of the magnet on the localization performance was marginal by applying the differential method compared with the absolute method.

### 3.7. Systematic Evaluation of the Non-Idealities of the Proposed Localization System

**Figure 7:**
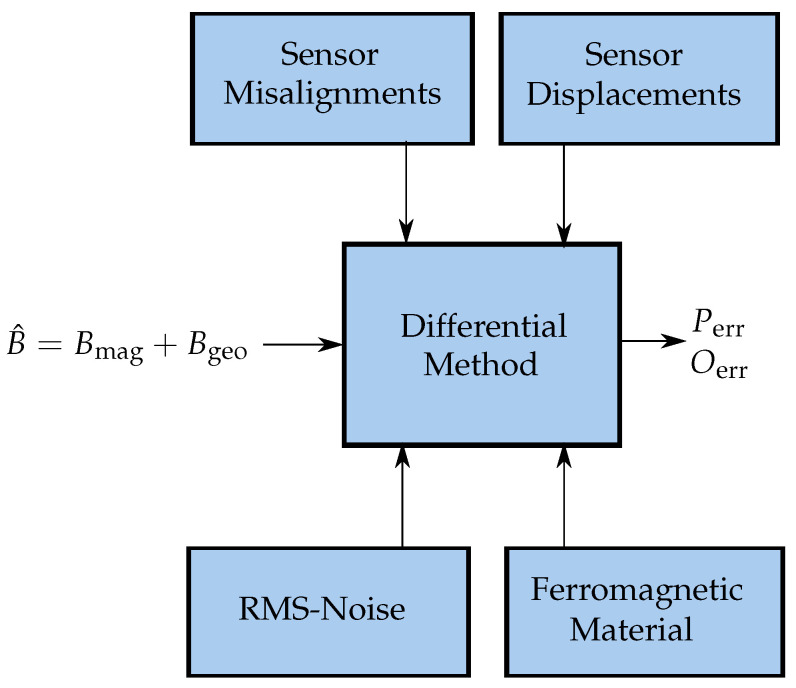
Block-diagram of various non-idealities influencing the proposed differential localization setup.

The performance of the differential localization method under the influence of various non-idealities on the localization setup was systematically evaluated. The considered influences are summarized in Figure 7. The geomagnetic flux density $B_{\mathrm{geo}}$ can be assumed as a homogeneous interference whose components are vectorially equal at all 12 sensors, as long as ideal conditions are applied. However, sensor misalignment and ferromagnetic material (soft magnetic disturbance) in the proximity of the magnetic sensors lead to fluctuation in the measured magnetic field $\hat{B}$. This also includes $B_{\mathrm{geo}}$, which is, therefore, not vectorially equal at each of the sensors anymore. Therefore, the geomagnetic field can no longer be assumed homogeneous, and thus, the performance of the differential localization method will suffer. When sensors are displaced, the orientation of the sensors and $B_{\mathrm{geo}}$ at the sensor positions will not change; however, the measured $\hat{B}$ will fluctuate since the distance from the magnet to the sensors changed. Additionally, the RMS-noise of the sensors will lead to fluctuation in the measured $\hat{B}$. The influence of these different non-idealities on the performance of the differential method was evaluated. For each scenario, four different orientations of the magnet (*x*-, *y*-, *z*-, and $\frac{1}{\sqrt{3}}(1,\;1,\;{1)}^{⊺}$-orientation) were applied while having the the position of the magnet fixed at ${\left(60,\;60,\;60\right)}^{⊺}$ mm. At first, each of the non-idealities was applied separately to the localization system. Subsequently, all considered disturbances were added simultaneously to evaluate the proposed system under more realistic conditions. Moreover, the different diameter-to-length ratios of our previous study [26] were considered. Finally, the localization performance was investigated when the system was rotated since this is crucial for a wearable localization system.

#### 3.7.1. Evaluation of Sensor Displacement

The localization of endoscopy capsules is based on a reference coordinate system (Figure 3). Therefore, the relative sensor positions on the three rings must be known for accurate localization of the capsule. In a real setup, sensor displacement due to manufacturing tolerance and mechanical displacement of the localization system has to be considered. Therefore, to investigate the stability of the localization system under this condition, the three components of each sensor position in the COMSOL simulation were displaced with uniformly distributed random values from ±1 mm to ±5 mm in steps of 1 mm.

#### 3.7.2. Evaluation of Sensor Misalignment

**Figure 8:**
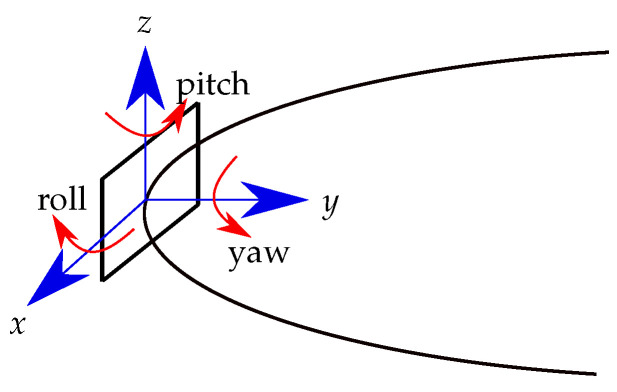
A representative sensor and its reference coordinate system (blue). The sensor is rotated around the three angles of roll, yaw, and pitch.

As the differential localization method is based on equally aligned sensor pairs (described in Section 3.3), the relative misalignment of those two sensors leads to vectorially unequal interference of the geomagnetic flux density, and thus, the geomagnetic flux density will not completely cancel out. For the investigation of the influence of misalignment, each sensor was randomly misaligned. This was done by applying a rotation matrix on each sensor reading in COMSOL. Thus, the sensor surface normal vector was rotated around the three axes according to the roll (*x*-axis), yaw (*y*-axis), and pitch (*z*-axis) angle (Figure 8). The sensor orientation was varied by adding uniformly distributed random values from ±1° to ±5° in steps of 1°.

#### 3.7.3. Evaluation of RMS-Noise of Magnetic Sensors

For the proposed differential localization method, an RMS-noise of 0.5 µT was applied in the COMSOL simulation on each sensor reading. In [32], we already investigated the influence of the RMS-noise of different amplitudes of magnetometers on the proposed differential method. It was revealed that an RMS-noise of 0.5 µT led to mean position and orientation errors below 1 mm and 1°, respectively. The magnetic flux density generated by a permanent magnet decays with approximately $1/R^{3}$; therefore, the impact of the RMS-noise on the measured *B* significantly depends on the distance from the magnet to a sensor. The influence of the RMS-noise on the measured flux density depending on the distance from the permanent magnet was investigated in this study. Hence, we measured the magnetic flux density *B* with respect to the distance from the proposed magnet and compared it with the RMS-noise and the simulated *B* while the geomagnetic flux density was calibrated. The distance from the magnet was adapted to the localization setup and was varied from 10 cm to 40 cm in steps of 10 cm. The sensor settings were chosen as described in Section 3.4, and the mean and standard deviation values for each evaluated distance were determined for 10 samples each time.

#### 3.7.4. Evaluation of the Ferromagnetic Material in the Proximity of the System

**Figure 9:**
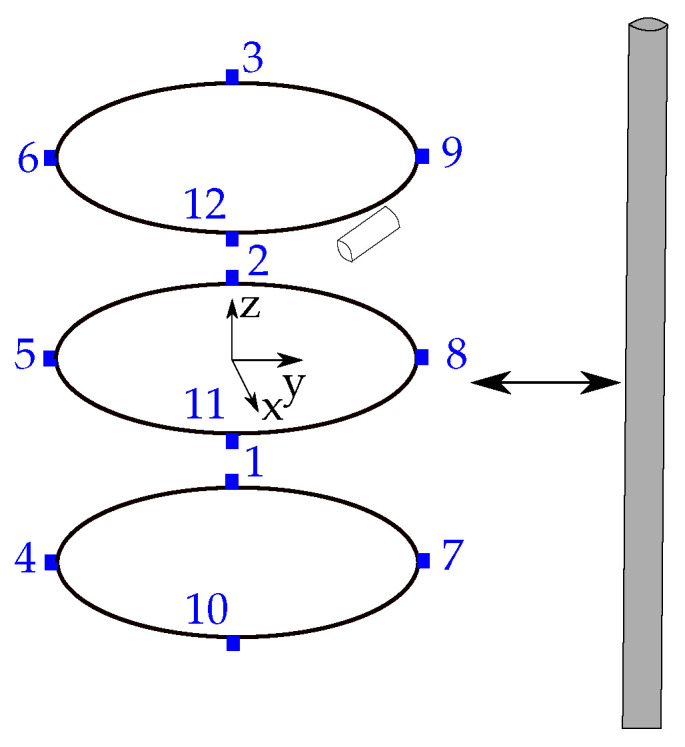
Iron cylinder in the proximity of the sensor array. The cylinder is *z*-orientated, whereas the displacement of the cylinder is in the *y*-direction.

By placing the ferromagnetic material in the proximity of the magnetic sensors, the geomagnetic field and the magnetic field of the permanent magnet used for the localization will be distorted. The differential method assumes that the geomagnetic field is vectorially equal at all sensors. Therefore, the ferromagnetic material will increase the localization error because it results in an inhomogeneous geomagnetic field. During the treatment of WCE, it is most likely that the magnetic sensors on the body surface will be in the proximity of the ferromagnetic material (e.g., a metal frame of a bed or heating element). To investigate this effect, a cylinder with a diameter of 5 cm and a height of 40 cm of linear isotropic magnetic material with a relative magnetic permeability of µ_r_ = 4000 was considered in the COMSOL simulations (Figure 9).

The *z*-orientated cylinder was placed in the proximity of the sensor rings. The distance from the cylinder surface to Sensors 7, 8, and 9 was varied from 50 cm to 1 cm in *y*-direction. In Section 3.4, the influence of a ferromagnetic heating element on the measured values of a sensor pair was experimental evaluated. Therefore, to study the effect of this difference in measured values, the resulting localization errors for the heating element with a distance of 50 cm away from Sensors 10, 11, and 12 was determined in simulations. The heating element was assumed as a full-block isotropic linear ferromagnetic material with µ_r_ = 4000. The dimensions and orientation were chosen as described in Section 3.4. The measured components of the geomagnetic flux density were applied in the simulations.

### 3.8. Evaluating All Considered Non-Idealities Simultaneously

As a next step, the differential localization method for capsule endoscopy was evaluated by considering all aforementioned non-idealities. For the sensor displacement and misalignment values, the manufacturing tolerance of the proposed system was considered simultaneously. Therefore, random displacement of sensor positions in the range of [−1, +1] mm for the *x*-, *y*-, and *z*-directions was applied on each of the 12 sensors. Additionally, the sensors were randomly misaligned in the range of [−1, +1]° with respect to roll-, yaw- and pitch-rotation. Moreover, a linear isotropic ferromagnetic cylinder in the proximity of the sensors (30 cm away from the setup) was added. Finally, an RMS-noise of 500 nT was added on each sensor reading. In our previous study [26], we applied different diameter-to-length ratios of the magnet and evaluated the resulting position and orientation errors. The results showed that the ratio had no significant influence on the localization performance. However, the localization system was evaluated under ideal conditions. Therefore, in this study, we varied this ratio again and applied all considered non-idealities.

### 3.9. Evaluation of Rotation of the Localization System under Non-Ideal Conditions

In [32], we compared the differential and absolute localization system under ideal conditions when the entire system was rotated around the *x*-, *y*-, and *z*-axes. For a wearable localization system for the application of WCE, the localization performance must be accurate during the entire diagnosis procedure. The displacement and misalignment of sensors on the sensor rings, as well as the RMS-noise cannot be prevented in a real localization setup. Therefore, the random values of Section 3.8 for the displacement, misalignment, and RMS-noise of the sensors were considered, and the entire system was rotated for different sizes of magnets. The evaluation procedure for the different rotations of the system was described in detail in [32].

## 4. Results

### 4.1. Experimental Validation of the Differential Geomagnetic Compensation Method

To evaluate the feasibility of the proposed differential method, experimental measurements were conducted. Table 1 summarizes the results of this experimental study.

At first, a reference measurement was conducted where the sensors were aligned and no ferromagnetic material was nearby. The measured difference in the magnetic flux density was not higher than 1.1 µT for that scenario. When Sensor 2 was displaced by 5 mm in the *x*-direction, the maximal distance in *B* was approximately the same with 1.3 µT. In contrast, when Sensor 2 was rotated by 5° around the *z*-axis, the maximal difference in *B* was increased by approximately a factor of three compared with the reference measurement. In the case of Sensor 2 being 50 cm next to a ferromagnetic heating element, the maximal difference between sensor 1 and Sensor 2 was 5.3 µT.

### 4.2. Reference Results under Ideal Conditions

Table 2 shows the results for the mean position and orientation errors of the four different applied orientations of the magnet under ideal conditions. It has to be noted that the position and orientation errors were constant while the localization system was rotated. The mean position and orientation errors were 0.05 mm and 0.05°, respectively. These results were proposed in our previous work [32] and optimized in [26].

### 4.3. Results for Systematic Evaluation of Non-Idealities of the Localization System

#### 4.3.1. Results for Sensor Displacement

**Figure 10:**
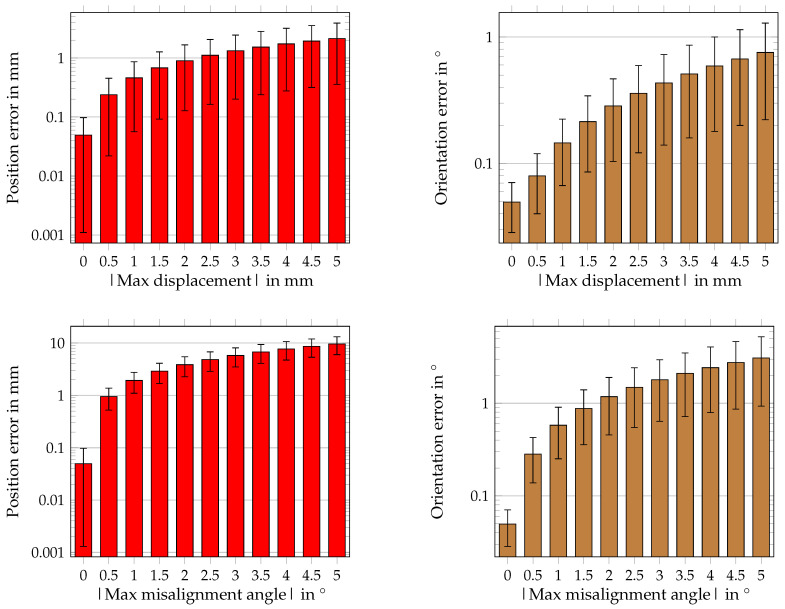
Mean position and orientation errors for different maximal random displacement and misalignment of sensors. The *y*-axis is in log-scale.

The mean position and orientation errors next to the standard deviations are depicted in Figure 10, for applying a random displacement on each sensor position. The position and orientation errors increased approximately linear for higher applied random displacement. The slope of the position error was approximately 0.4 mm per maximal 1 mm of random displacement, whereas the slope of the orientation error was 0.16° per maximal 1 mm of random displacement. This led to maximal position and orientation errors of approximately 2.1 mm and 0.8°, respectively. The four different orientations of the magnet led to STD values that were of the same order as the corresponding position and orientation errors. The values of the STD also increased linearly for higher random displacement.

#### 4.3.2. Results for Sensor Misalignment

In this section, the influence of the misalignment of the sensors on the localization performance is investigated. Figure 10 shows the resulting mean position and orientation errors next to the STD values by applying random misalignment. The mean position and orientation errors increased approximately linearly for higher applied random misalignment values. The slope of the position error was approximately 2 mm per a maximal 1° random misalignment, whereas the slope of the orientation error was 0.6° per a maximal 1° random misalignment. This led to maximal position and orientation errors of approximately 10 mm and 3°, respectively. The influence of the four different orientations of the magnet is represented by the STD values. The values of the STD also increased linearly for higher random misalignment. However, compared with the STD values by applying random displacement, the ratio of the mean position and orientation errors and the corresponding STD value was smaller.

#### 4.3.3. Results for the RMS-Noise of Sensors

The influence of an RMS-noise of 500 nT on each sensor measurement was investigated in [32]. The resulting position and orientation errors for the four applied orientations of the magnet are summarized in Table 3.

The position and orientation errors were highest when the magnet was $\frac{1}{\sqrt{3}}(1,\;1,\;{1)}^{⊺}$-orientated with 1.36 mm and 0.45°, respectively. The lowest position error was 0.61 mm for a *y*-oriented magnet, whereas the minimum orientation error was 0.04° for a magnet with a *z*-orientation. Overall, the mean values and STD for the position and orientation errors were 0.85 ± 0.30 mm and 0.31 ± 0.16°, respectively. The influence of the different magnet orientations was slightly higher than for the ideal scenario since the STD values were increased by one order in magnitude.

**Figure 11:**
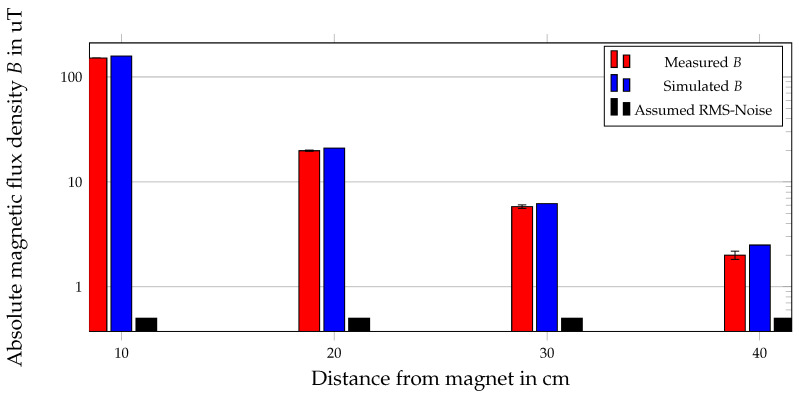
Absolute magnetic flux density *B* depending on the distance from the magnet. The measured magnetic flux density and its standard deviation (STD) are compared with the simulated one from COMSOL. Moreover, the assumed RMS-noise is depicted.

In Figure 11, the absolute magnetic flux density *B* with respect to the distance from the magnet is shown.

The magnetic flux density of the COMSOL simulation was compared with that measured by an LSM303D. The ratio of the measured and the simulated *B* was approximately 0.96 for a distance up to 20 cm. For a distance of 30 cm, the ratio was approximately 0.94, and for a distance of 40 cm, it was 0.8. Besides, for a distance up to 20 cm, the measured and simulated *B* were at least two orders of magnitude higher than the assumed RMS-noise, while for a distance of approximately 25 cm and higher, it was only one order of magnitude higher than the assumed RMS-noise.

#### 4.3.4. Results for Ferromagnetic Material in the Proximity of the System

**Figure 12:**
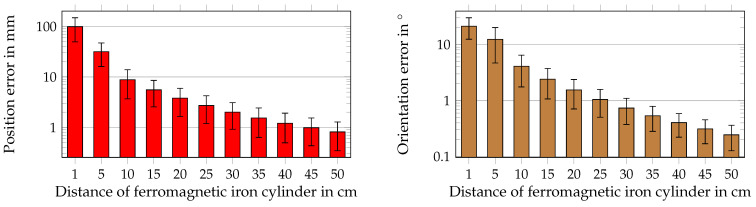
Position and orientation errors for different distances from the localization system to an iron cylinder.

Figure 12 shows the influence of ferromagnetic material, depending on the distance from the sensor setup, on the localization performance.

For a distance below 10 cm, the mean position error was up to 100 mm, whereas it was around 1 mm for a distance of 40 cm. Moreover, for a distance higher than 5 cm, the slope of the position error was significantly reduced and started to flatten for a distance higher than 30 cm. The orientation error was up to approximately 20° for a distance smaller than 5 cm. At distances higher than 25 cm, the orientation error was smaller than 1°. The observed curve flattening of the position error was less severe for the orientation error. Overall, the position and orientation errors significantly decreased with the sensor array being further away from the ferromagnetic material. The values of the STD followed the same trend as the mean position and orientation errors. However, compared with the STD values by applying random displacement and misalignment, the ratio of the mean position and orientation errors and the corresponding STD value was smaller. Besides, the localization errors for a heating element at a distance of 50 cm next to Sensors 10, 11, and 12 are summarized in Table 4. The mean position error was 0.38 mm approximately and, thus, reduced by a factor of two compared with the position error for a ferromagnetic cylinder at a distance of 50 cm, while the orientation error was in good agreement with 0.26°.

#### 4.3.5. Results for the Combination of all Considered Non-Idealities and the Variation of the Magnet Length on the Localization System

**Figure 13:**
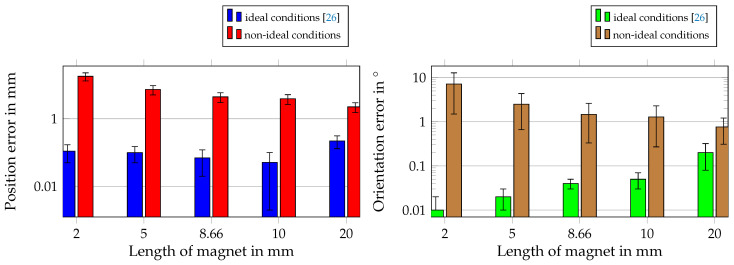
Comparison of localization performance under non-ideal conditions with performance under ideal conditions [26] for different applied magnet sizes.

The results for the combination of all considered non-idealities and for different magnet lengths are shown in Figure 13.

The results of this study were compared with those of [26], where ideal conditions were applied. For non-ideal conditions, the highest mean position and orientation errors occurred with the shortest magnet of length 2 mm with approximately 16 mm and 7°, respectively. The smallest errors were approximately 2.5 mm and 1°, respectively, for the longest magnet of length 20 mm. The localization performance was enhanced when a longer magnet was used. However, for magnets of a length 5 mm and longer, the margins of errors overlapped. In contrast, when ideal conditions were applied, the position and orientation errors were highest for a magnet of a length of 20 mm, and the orientation error increased with the magnet length.

#### 4.3.6. Results for the Rotation of the System under Non-Ideal Conditions

**Figure 14:**
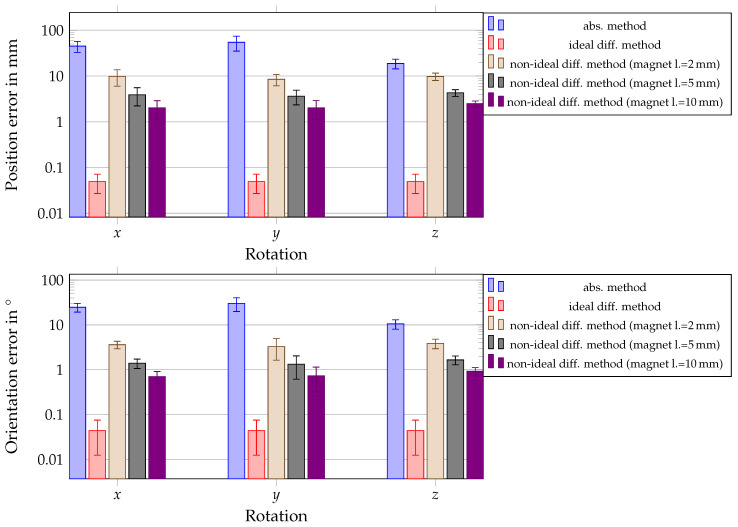
Comparison of the absolute method and differential method by applying displacement, misalignment, and RMS-noise for rotations of the system around the −x-, −y- and −z-axes and for different sizes of magnets.

In Figure 14, the position and orientation errors next to the STD values for all three rotations of the entire localization system for the different applied scenarios are shown.

The bar graph compares the absolute method with the differential method. For the absolute method, a magnet of a length of 10 mm was applied. Since the results in [32] revealed that the localization performance of the absolute magnetic localization system was not reliable when the entire system was rotated under ideal conditions, non-ideal conditions, as well as different magnets were not applied. For the differential method, different magnet lengths were applied. The results revealed that the differential method decreased the position and orientation errors at least by one order in magnitude. The localization performance varied significantly for the different rotations of the system when the absolute localization method was applied. In contrast, the margins of position and orientation errors for applying the differential method overlapped for the three different applied rotations of the system, and thus, the localization performance was significantly more stable. Moreover, overlap in the margins of error for magnets longer than 2 mm can be observed. The mean position and orientation errors were below 10 mm and 4°, respectively, when the system was rotated even for a magnet of a length of 2 mm. For a magnet of a length of 10 mm, the mean position and orientation errors were approximately 2 mm and 1°, respectively. The performance was constant for all rotations for the differential method under ideal conditions.

## 5. Discussion

In this study, the differential geomagnetic compensation method was experimentally validated by comparing the measured values of the geomagnetic flux density of two LSM303D sensors with a distance of 40 cm to each other. The results revealed that the difference between the measured values of the two sensors was not higher than 1.3 µT when the two sensors were aligned and there was no nearby ferromagnetic material. Therefore, the measured difference was approximately of the same order as the assumed RMS-noise, and the assumption of the homogeneous geomagnetic flux density for the relatively small localization setup (33 cm × 40 cm × 20 cm) was valid. Moreover, a rotation of Sensor 2 by approximately 5° resulted in a maximal difference in the measured *B* of approximately 3 µT. By comparing the magnetic flux density of a permanent magnet (Figure 11) with this difference, it was concluded that the impact of misalignment significantly depended on the distance of the corresponding sensor to the magnet. Hence, the maximal difference of 3 µT was approximately of the same order as *B* of a permanent magnet for a distance from the magnet to a sensor larger than 20 cm. The difference in the measured values of the two sensors was highest for a ferromagnetic heating element in the proximity of the two sensors. Sensor 2 was closer to the heating element; thus, its magnetic flux density was significantly more distorted than that of Sensor 1. The maximal difference in *B* for that scenario was approximately 5 µT. Therefore, the ferromagnetic material in the proximity of the sensor setup yielded a high potential of error for the proposed differential method. This experimental study revealed that the proposed differential method is feasible; however, the sensors must be aligned and ferromagnetic material in the proximity of the setup avoided.

Moreover, a simulation-based study was conducted, where the non-idealities of all sensors were considered and the localization performance for the different scenarios was determined. Besides, the localization performance for different magnet lengths was evaluated. Finally, the proposed localization system was rotated to consider daily life situations of a patient. In the following, the results of the systematic evaluation of the system are discussed.

First, the sensor positions were randomly displaced with different maximal displacement values. The position and orientation errors increased approximately linearly with maximal random displacement. Compared to the results for ideal conditions, the mean and STD values of the position and orientation errors were increased by two orders in magnitude. Therefore, the margins of error were significantly increased when different magnet orientations were applied and the sensors randomly displaced. The random displacement of sensors had the smallest impact on the differential method. Thus, the maximal position and orientation errors by applying random displacement were approximately four times smaller than those of the random misalignment. The maximal position and orientation errors for the ferromagnetic material in the proximity of the system were even about 50 and 25 times higher than those of the random displacement. This is because the geomagnetic flux density can be assumed as homogeneous for the relatively small localization system, which was also shown in the results of the experimental study of Section 4.1. Therefore, the elimination of the geomagnetic flux density at the sensors still works if sensors are displaced. The resulting localization errors were due to the unknown sensor positions.

Subsequently, the sensors were randomly misaligned. The position and orientation errors increased linearly for random misalignment of the sensors. The slope of the error curves was steeper than those of the random displacement of sensors. The results revealed that the orientation of the sensors significantly affects the localization performance since the mean and STD values of the position and orientation errors were increased by up to two orders in magnitude compared with the results for ideal conditions. Because the measured values of sensors corresponding to a pair were subtracted, misalignment of those two sensors led to a change in the components of the geomagnetic flux density measured at the sensors. This was also validated experimentally. The maximal measured difference in *B* of the two considered sensors was approximately 3 µT. Therefore, for a practical implementation, it is recommended to keep the misalignment of a pair of sensors below 5°.

Moreover, an RMS-noise of 500 nT was applied to each sensor measurement, and thus, the mean and STD values of the position and orientation errors were increased by one order in magnitude. Therefore, the influence of the RMS-noise was significantly smaller compared with the other evaluated non-idealities. However, it should be noted that the mean distance from the magnet to the sensors was 21.8 cm ± 5.9 cm. In Section 4.3.3, it was revealed that the impact of the RMS-noise significantly depended on the distance from a sensor. Since the mean distance from the magnet to the sensors was approximately 22 cm, the position and orientation errors were relatively small. Compared with the planar sensor array proposed in [25], our localization setup yielded the advantage of higher spatial diversity, and therefore, the mean distance from the magnet to the sensors was relatively stable, while it significantly depended on the magnet position for a planar array. The measured and the simulated *B* were in good agreement; for a distance of 40 cm, the RMS-noise had a significant impact on the measured values, and therefore, the deviation from the simulated *B* increased. Overall, the simulated *B* was higher than the measured one, and this was due to the magnetization of 1150 kA/m, which was assumed in the COMSOL simulations. The real magnetization of the magnet was determined by the manufacturing tolerance.

Next, an iron cylinder and heating element were placed in the proximity of the sensor rings. The results showed that a ferromagnetic cylinder with a distance below 30 cm increased the position and orientation errors up to three orders in magnitude. For an iron cylinder at a distance of 50 cm, the position and orientation errors were below 1 mm and 0.3°, respectively. In contrast, the position error was significantly lower with approximately 0.4 mm in the case that a heating element was placed 50 cm next to the setup. This can be explained by the theory of the demagnetization factor [34]. The cylinder was *z*-oriented and had a smaller cross-section than the heating element. However, the length in the *z*-direction was approximately the same for the heating element and cylinder. Therefore, compared with the heating element, the cylinder had a smaller demagnetization factor and, thus, was more strongly magnetized by the geomagnetic field. Therefore, the cylinder led to a higher distortion of the geomagnetic field. The effect of the ferromagnetic material on the proposed differential method was also experimentally investigated. A representative sensor pair was placed at a distance of 50 cm next to a heating element. The difference of the measured values of the two LSM303D sensors was up to 5.3 µT. Besides, we evaluated *B* generated by a permanent magnet and compared it with the difference in measured values of the sensor pair. It was concluded that the difference of several µT would have a significant impact on the localization performance when the distance from the corresponding sensor to the magnet was larger than 20 cm. In a real-world application, the localization system must be kept away by at least 30 cm from the ferromagnetic material. Scenarios like driving a car or taking an elevator are not suitable for the proposed method as long as the magnetic sensors are not calibrated for these involvements. Overall, the ferromagnetic material in the proximity of the localization system yielded potentially the highest localization errors compared to the other evaluated cases. In the presence of magnetic fields, a ferromagnetic material like iron leads to soft magnetic disturbance, which is inhomogeneous. Therefore, it disturbs the geomagnetic flux density, as well as the measured flux density from the magnet. Since the differential localization method is only suitable for eliminating interference whose components are equal for all sensors, a ferromagnetic material leads to high errors. Moreover, the distance from a sensor to ferromagnetic objects significantly affects the order of magnitude of the localization error. At first sight, it seems unexpected that the localization error in the case of the nearby heating element or cylinder was smaller than for the misalignment of sensors since the difference in the measured values of the representative sensor pair was higher for the heating element than for the misalignment of 5°. However, it should be noted that in the simulation-based systematic study, all 12 sensors were randomly misaligned, and in the case of the nearby heating or ferromagnetic cylinder, only the measured values of the three closest sensors were significantly distorted.

As a next step, all aforementioned distortions were considered simultaneously, and the length of the magnet was varied to evaluate the system under more realistic conditions. In [26], we already varied the diameter-to-length ratio; however, ideal conditions were applied, and the ratio had no significant influence on the localization performance. Therefore, we assumed that a magnet of length 2 mm would lead to a sufficient localization performance. However, under non-ideal conditions, the length of the magnet should be at least 5 mm to achieve a sufficient localization performance.

In the last step of the systematical evaluation of the proposed differential localization method, the entire system was rotated to test the capability of the system for daily life situations of a patient. The absolute localization method results were compared with those of the differential method under ideal and non-ideal conditions. Moreover, the magnet length was varied when the differential method was applied under non-ideal conditions. The results showed once more that a magnet of length 2 mm led to high position and orientation errors of approximately 10 mm and 3°, respectively, when non-ideal conditions were applied on the differential method. For a magnet of a length of 5 mm and above, the position and orientation errors were significantly smaller, and the margins of error overlapped. Therefore, for a real setup, it is suggested to use a magnet with a diameter of 10 mm and at least a length of 5 mm to achieve a reliable localization performance for non-ideal conditions and the rotation of the system. Overall, these results demonstrated that the proposed system can achieve a reliable localization accuracy for a wearable localization system for capsule endoscopes. The results of this systematic performance evaluation of our proposed system will significantly aid in designing an experimental setup of the proposed differential method.

### Comparison of State-of-the-Art Localization Methods for Capsule Endoscopes

In the following, the proposed differential method is compared with state-of-the-art localization methods for capsule endoscopes. The localization accuracy of the corresponding methods is summarized in Table 5.

In the literature, quasi-static and static magnetic localization methods for WCE have been proposed. The differential method is assigned to the latter and, therefore, shall be firstly compared to static methods. Shao et al. (2019) [35] proposed a method for geomagnetic compensation for capsule endoscopes. Herein, two magnetic sensors were fixed on the chest and back of a patient in addition to the sensor array around the abdomen. Due to the distance between the sensor array and the additional sensors, it was assumed that they measured only the geomagnetic flux density. Therefore, by subtracting these measured values from the measured values at the sensor array, the geomagnetic flux density was eliminated. The localization system was rotated, and the achieved mean position and orientation errors were approximately 10 mm and 12°, respectively. However, the results revealed that especially the orientation error deviated approximately in the range of 10° for different rotations of the system. This could result from the additional sensors, which were mounted on the chest and back, and therefore, their orientation was not stable. The results of our study showed that the proposed differential localization system is significantly more robust and accurate for the application of a wearable localization system if the magnet length is at least 5 mm.

Dai et al. (2019) proposed another geomagnetic compensation method for wireless capsule endoscopy [25]. In Dai’s approach, an inertial measurement unit (IMU) was used together with a magnetic sensor array for the localization of a permanent magnet. The IMU data were used to estimate the posture of the magnetic array and, therefore, separate the geomagnetic flux density from the magnetic flux density generated by the permanent magnet when the system was rotated. Thereby, position and orientation errors of 3.89 mm and 5.5°, respectively, were experimentally achieved. The position error of their proposed method is comparable to our proposed differential method for a magnet of a length of 5 mm. However, for a length of 10 mm, our system’s performance was significantly better. The results of our study, moreover, showed that the orientation error was significantly lower and stable for our proposed system. Furthermore, an additional IMU usage for geomagnetic compensation has to be tested for the average diagnosis duration of WCE, which is around 8 h. The influence of the drift error was not fully considered in Dai’s study. Moreover, orientation estimation with an IMU and without the usage of a magnetometer is limited in accuracy.

A coil-based geomagnetic compensation method was proposed by Shimizu et al. (2020) [18]. Herein, an endoscopy capsule was equipped with magnetic and acceleration sensors for estimating the orientation and position of a capsule. A coil as a magnetic field source was integrated into a wearable neck corset for the patient. By switching the coil on and off, the geomagnetic field was canceled in the measured values. The achieved position and orientation errors were 10 mm and 5°, respectively. Compared with our proposed method, this method performed significantly worse. Moreover, the batteries of a capsule endoscope have a limited capacity. Therefore, the batteries cannot provide the integrated sensors and camera with power for the entire diagnosis procedure. Furthermore, only an acceleration sensor was used for estimating the posture of the capsule. Thus, this approach will face the same problem with drift error over time as Dai’s approach. This method should be tested within an appropriate time interval of 8 h.

On the other hand, quasi-static magnetic localization methods are based on alternating-current signals in the kilohertz range generated by a coil, and thus, no geomagnetic compensation method is required. The magnetic field generated by a coil outside the body was sensed by receiving coils [15,19] integrated into the capsule. In this way, position and orientation errors below 3 mm [15,19] and 0.2° [19] were reported. The results showed that quasi-static magnetic localization methods are competitive with static methods. However, since the magnetic field is measured inside the capsule, the number of sensors/receiving coils is inherently restricted. Moreover, Yang et al. [19] used a single uni-axial receiving coil; therefore, the localization performance significantly depended on the orientation of the capsule.

Finally, the proposed method was compared with RF-based localization methods for the application of WCE. The RF-based localization methods utilize the transmission of a video stream for the localization. Therefore, the frequency range of these methods is in the megahertz to gigahertz range. Multiple antennas were arranged outside the body for localization to sense the RF signal from the capsule. The achieved position error by Khan et al. [12], as well as Barbi et al. [36] by using RF-based methods was for both studies approximately 10 mm. The main source of error for the RF-based methods is the inhomogeneous permittivity of human tissue, which differs for different patients.

For a reliable diagnosis of the gastrointestinal tract, the capsule endoscopy must be accurately tracked for a relatively long duration of approximately 8 h. By considering the size of a capsule, which is approximately 32 mm × 12 mm, the position error should be at least smaller than 5 mm to resolve the capsule. Moreover, the application of WCE is intended to enable the patient to leave the hospital during the diagnosis. Therefore, the use-cases of WCE are daily life situations of a patient. Hence, a localization system for WCE should be in particular wearable and robust. The proposed differential method fulfills these requirements and is therefore the next step towards precise localization of capsule endoscopes within the daily life of a patient.

## 6. Conclusions

In this study, the feasibility of the proposed differential method was experimentally validated since the measured difference between two equally orientated sensors was approximately of the same order as the root-mean-squared sensor noise. The rest of the study was simulation based. The influence of non-idealities on our proposed differential method for localizing capsule endoscopes was systematically investigated. Hence, the influence of sensor displacement, misalignment, and root-mean-squared noise on the localization performance was investigated individually. Moreover, the influence of ferromagnetic material in the proximity of the proposed system was considered. The results revealed that ferromagnetic material and misalignment of sensors lead to the highest localization errors. Moreover, to test the localization performance for daily life situations, the entire system was rotated when the sensors were displaced, misaligned, and RMS-noise was added to each measurement. Additionally, the magnet length was varied. The results revealed that the mean position and orientation errors were increased from below 0.1 mm and 0.1° to several millimeters and degrees, by applying non-ideal conditions. For a magnet of length 5 mm, the position and orientation errors were approximately 4 mm and 1°, respectively, and for a length of 10 mm approximately 2 mm and 1°, respectively. Compared with state-of-the-art geomagnetic compensation methods for the localization of capsule endoscopes, the proposed localization method was significantly more robust and accurate for a magnet length of 10 mm. For a magnet of length 5 mm, the localization performance was still reliable and competitive with state-of-the-art methods.

Moreover, the results showed that the localization system must be kept away from ferromagnetic materials as much as possible and the position and orientation errors depend on the geometry and orientation of the ferromagnetic object, as well as the relative position of the capsule within the localization setup. Therefore, use-cases like driving a car or taking an elevator or situations in which the sensor setup is misaligned are critical for the proposed method. In contrast, the displacement of sensors has no significant impact on the localization performance. Furthermore, it was concluded that the impact of the root-mean-squared noise of magnetic sensors significantly depends on the distance from the magnet to a sensor. This knowledge will aid in designing an optimal localization setup for real measurements.

## Figures and Tables

**Table 1:** Comparison of the three measured values at the two LSM303D. the absolute difference between the corresponding measured components is given.

	$B_{x}$ (µT)	$B_{y}$ (µT)	$B_{z}$ (µT)
Reference measurement			
Sensor 1	−5.2	−19.5	−43.8
Sensor 2	−6.3	−19.8	−43.4
Difference	1.1	0.3	0.4
*x*-Displacement of Sensor 2 + 5 mm			
Sensor 1	−5.2	−19.5	−43.8
Sensor 2 displaced	−6.5	−19.2	−43.5
Difference	1.3	0.3	0.3
Rotation around the *z*-axis of Sensor 2 + 5°			
Sensor 1	−5.2	−19.5	−43.8
Sensor 2 rotated	−8.1	−19.0	−43.5
Difference	2.9	0.5	0.3
Heating element approximately 50 cm next to Sensor 2			
Sensor 1	−8.7	−18.3	−42.7
Sensor 2	−7.2	−15.6	−37.4
Difference	1.5	2.7	5.3

**Table 2:** Position $P_{\mathrm{err}}$ and orientation $O_{\mathrm{err}}$ errors and their mean value and standard deviation (STD) for the differential method for the four different orientations of the magnet under ideal conditions [32].

Orientation of the Magnet:	*P*_err_ in mm	*O*_err_ in °
$(1,\;0,\;{0)}^{⊺}$	0.01	0.07
$(0,\;1,\;{0)}^{⊺}$	0.05	0.07
$(0,\;0,\;{1)}^{⊺}$	0.03	0.01
$\frac{1}{\sqrt{3}}(1,\;1,\;{1)}^{⊺}$	0.14	0.06
Mean value and STD	0.05 ± 0.05	0.05 ± 0.02

**Table 3:** Position and orientation errors for a 500 nT RMS-noise.

Orientation of the Magnet:	*P*_err_ in mm	*O*_err_ in °
$(1,\;0,\;{0)}^{⊺}$	0.67	0.42
$(0,\;1,\;{0)}^{⊺}$	0.61	0.34
$(0,\;0,\;{1)}^{⊺}$	0.75	0.04
$\frac{1}{\sqrt{3}}(1,\;1,\;{1)}^{⊺}$	1.36	0.45
Mean value and STD	0.85 ± 0.30	0.31 ± 0.16

**Table 4:** Position $P_{\mathrm{err}}$ and orientation $O_{\mathrm{err}}$ errors and their mean value and standard deviation (STD) for the differential method for the four different orientations of the magnet. A heating element at a distance of 50 cm was placed next to the setup.

Orientation of the Magnet:	*P*_err_ in mm	*O*_err_ in °
$(1,\;0,\;{0)}^{⊺}$	0.4	0.4
$(0,\;1,\;{0)}^{⊺}$	0.2	0.03
$(0,\;0,\;{1)}^{⊺}$	0.4	0.3
$\frac{1}{\sqrt{3}}(1,\;1,\;{1)}^{⊺}$	0.5	0.3
Mean value and STD	0.38 ± 0.11	0.26 ± 0.13

**Table 5:** Comparison of state-of-the-art localization methods for capsule endoscopes. For the proposed differential method, non-ideal conditions, rotation of the entire system, and a magnet of length 10 mm were applied.

Method:	Year	*P*_err_ (mm)	*O*_err_ (°)
**Static Magnetic:**			
Proposed differential method (simulations) [26]	2020	2	1
Shao et al. [35]	2019	10	12
Dai et al. [25]	2019	5	6
Shimizu et al. [18]	2020	10	5
**Quasi-static Magnetic:**			
Islam et al. [15]	2018	3	-
Yang et al. [19]	2020	2	0.2
**RF-based:**			
Barbi et al. [36]	2019	10	-
Geng et al. [13] (simulations)	2015	<10	-

## Data Availability

Not applicable.

## Abbreviations

The following abbreviations are used in this manuscript:

GIT	Gastrointestinal tract
IMU	Inertial measurement unit
RF	Radio-frequency
RMS	Root-mean-square
RSS	Received signal strength
STD	Standard deviation
TOA	Time of arrival
WCE	Wireless capsule endoscopy
